# SPOCK1 promotes the invasion and metastasis of gastric cancer through Slug‐induced epithelial‐mesenchymal transition

**DOI:** 10.1111/jcmm.13357

**Published:** 2017-09-22

**Authors:** Dehu Chen, Haihua Zhou, Guiyuan Liu, Yinghai Zhao, Gan Cao, Qinghong Liu

**Affiliations:** ^1^ Department of General Surgery Taizhou People's Hospital The Fifth Affiliated Hospital of Nantong University Taizhou Jiangsu China

**Keywords:** SPOCK1, gastric cancer, epithelial‐mensenchymal transition

## Abstract

Metastasis is a crucial impediment to the successful treatment for gastric cancer. SPOCK1 has been demonstrated to facilitate cancer metastasis in certain types of cancers; however, the role of SPOCK1 in the invasion and metastasis of gastric cancer remains elusive. SPOCK1 and epithelial‐mesenchymal transition (EMT)‐related biomarkers were detected by immunohistochemistry and Western blot in gastric cancer specimens. Other methods including stably transfected against SPOCK1 into gastric cancer cells, Western blot, migration and invasion assays *in vitro* and metastasis assay *in vivo* were also performed. The elevated expression of SPOCK1 correlates with EMT‐related markers in human gastric cancer tissue, clinical metastasis and a poor prognosis in patients with gastric cancer. In addition, knockdown of SPOCK1 expression significantly inhibits the invasion and metastasis of gastric cancer cells *in vitro* and *in vivo*, inversely, SPOCK1 overexpression results in the opposite effect. Interestingly, SPOCK1 expression has no effect on cell proliferation *in vitro* and *in vivo*. Regarding the mechanism(s) of SPOCK1‐induced cells invasion and metastasis, we prove that Slug‐induced EMT is involved in SPOCK1‐facilitating gastric cancer cells invasion and metastasis. The elevated SPOCK1 expression is closely correlated with cancer metastasis and patient survival, and SPOCK1 promotes the invasion and metastasis of gastric cancer through Slug‐mediated EMT, thereby possibly providing a novel therapeutic target for gastric cancer.

## Introduction

Gastric cancer is the fifth most common malignancy and is the third leading cause of cancer‐related deaths worldwide 1, 2. Considering the high probability of metastasis and recurrence and a deficiency of effective therapeutic strategies for patients with advanced gastric cancer during past decades, patients are much more susceptible to a poor prognosis even after a comprehensive therapy 3. Additionally, the molecular mechanisms responsible for the invasion and metastasis of gastric cancer remain poorly characterized. Therefore, identification of novel metastases‐related genes and elaboration of the underlying mechanism(s) may provide potential targets for anti‐cancer metastasis treatment.

Recent investigations have revealed that cancer cell activation of EMT contributes to cell invasion and metastasis in multiple cancers 4, 5. EMT is commonly known as a process of tightly connected epithelial cells transdifferentiated into motile mesenchymal cells, which is accompanied by the down‐regulation of epithelial cell junction protein, such as E‐cadherin, and the up‐regulation of mesenchymal markers such as Vimentin. Inversely, mesenchymal‐epithelial transition (MET) indicates the reverse process 6. As already reported, the switch in EMT process is performed by transcription factors, including the Snail family members Snail1 (Snail) and Snail2 (also addressed as Slug), as well as certain signalling pathways, containing receptor tyrosine kinases (RTKs), TGFβ, WNT, HIF1α and STAT3 signalling cascade, response to extracellular signals 4, 7.

Sparc/osteonectin, cwcv and kazal‐like domain proteoglycan 1 (SPOCK1), initially identified in human testes, encodes a Ca^2+^‐binding matricellular glycoprotein belonging to the secreted protein acidic and rich in cysteine (SPARC) family 8. Members of SPARC family share a similar structure, which is constitutive of N‐terminus, follistatin‐like domain and C‐terminus, and function in cell proliferation, migration and apoptosis in certain types of cancer 9, 10. In consideration of structural similarity between SPOCK1 and SPARC, it is interesting to note that SPOCK1 plays a crucial role in cancer cell invasion in oesophageal squamous cell carcinoma 11, colorectal cancer 12 and gallbladder cancer 13, which indicates that SPOCK1 may be a novel gene of interest, involved in the invasion and metastasis of cancer cells. However, in the light of our present knowledge, the function of SPOCK1 in the gastric cancer metastasis remains uncharacterized, and even less is known about the underlying mechanism responsible for SPOCK1‐mediated cancer progression.

Therefore, we investigated the role of SPOCK1 in gastric cancer metastasis and its potential mechanism(s). We demonstrated a significant positive correlation between high expression of SPOCK1 in primary lesion and poor prognosis in patients with gastric cancer, and a conclusion that SPOCK1 contributed to the invasion and metastasis of gastric cancer *via* Slug‐mediated EMT.

## Materials and methods

### Patient samples

All of the methods were approved by the Ethics Committee of the Fifth Affiliated Hospital of Nantong University and were performed in accordance with the approved guidelines and regulations. Primary lesion and corresponding noncancerous gastric tissue were obtained from 102 patients with gastric adenocarcinoma who underwent radical gastrectomy without preoperative treatment from 2011 to 2012, at the Department of General Surgery of our hospital. Among them, fresh tissues of 30 cases were also evaluated by Western blot for SPOCK1 protein. Primary lesion and para‐carcinoma tissue, confirmed by routine pathologic examination, were embedded in paraffin blocks for immunohistochemical stainings. Preoperative informed consent was obtained from all patients.

### Immunohistochemistry

Immunohistochemistry (IHC) analysis was carried out as described previously 14. Briefly, sections were deparaffinized, dehydrated and heat‐treated for antigen retrieval. Then, sections were blocked by hydrogen peroxide and blocking serum, followed by the following antibodies overnight: SPOCK1 pAb (1:100, Proteintech, Chicago, USA), E‐cadherin mAb (1:200, CST, Massachusetts, USA), Slug mAb (1:200, CST, Massachusetts, USA) and Vimentin (1:100, CST, Massachusetts, USA). Afterwards, sections were incubated with biotin‐conjugated anti‐IgG serum (Boster, China) and an SABC solution according to the product description. Finally, sections were observed through Diaminobenzidine (DAB) (Boster, China) incubation and scored under light microscope. Immunohistochemical scoring was assessed in accordance with the previous report 14. The percentage of staining cells was scored as 0 for 0–5%; 1 for 6–25%; 2 for 26–50% and 3 for 50–100%. The staining intensity was scaled as 0 for negative; 1 for weak intensity; 2 for moderate intensity and 3 for strong intensity. The sum scores ≥3 points were considered as positive, while the sum scores <3 points were regarded as negative.

### Cells and cell culture

The human gastric cancer cell lines (AGS, SNU216, SGC7901, MKN45, MGC803 and KATO‐III) and normal gastric epithelial GES‐1 cells were purchased from the Type Culture Collection of the Chinese Academy of Sciences (Shanghai, China). KATO‐III cells were cultured in 80% IMDM (ATCC, Virginia, USA) containing 20% foetal bovine serum (FBS) (Gibco, California, USA). The other cell lines were maintained in RPMI‐1640 medium replenished with 10% FBS. All cells were cultured in a humidified atmosphere of 37°C containing 5% CO_2_.

### Western blot analysis

Western blot analysis was carried out in line with standard procedures as described previously 14. Basically, proteins from tissues or cell lysates were separated by the sodium dodecyl sulphate–polyacrylamide gel electrophoresis (SDS‐PAGE), transferred onto polyvinylidene difluoride (PVDF) membranes (Millipore, Massachusetts, USA) and incubated with the following primary antibodies: SPOCK1 mouse pAb (1:500, Abcam, Cambridge, UK), E‐cadherin rabbit mAb (1:1000, CST, Massachusetts, USA), Snail rabbit pAb (1:1000, Abcam, Cambridge, UK), Slug rabbit mAb (1:1000, CST, Massachusetts, USA), Vimentin rabbit mAb (1:1000, CST, Massachusetts, USA), GAPDH mAb‐HRP (1:5000, Bioworld Technology, Minnesota, USA), followed by secondary antibody. Reactive bands were visualized using the enhanced chemiluminescence detection kit (Thermo scientific, Massachusetts, USA).

### Lentivirus infection

The short hairpin RNA (shRNA) oligonucleotide sequence for specifically targeting human SPOCK1 or Slug gene mRNA was designed using the RNAi designer. A negative sequence was employed as a control. The sequences of sh‐SPOCK1 and sh‐Slug were 5′‐GUAAUGAGGAGGGCUAUUA‐3′ 15 and 5′‐GAGGAAAGACTACAGTCCAAGTT‐3′, respectively. The lentivirus with SPOCK1‐gene was produced by cotransfection of 293T cells with Lipofiter, and transfected into SGC7901 and SNU216 cells, while Slug gene transfection for AGS cells. The human full‐length SPOCK1 cDNA was inserted into GV143 expression vector (Genechem, Shanghai, China) and transfected into AGS cells. AGS cells transfected with empty vector were employed as control. Following transfection, puromycin‐resistant cells were selected for subsequent studies. The protein expression of SPOCK1 was assessed by Western blot analysis.

### Wound‐healing assay

Cells were cultured in a 6‐well plate. A cell‐free wound was created using a 10 μl plastic tip. The process of cells migration into the wound area was imaged at 0 hr and 48 hrs time‐points. The wound healing = (0 hr width−48 hrs width)/0 hr width × 100% 14.

### Cell invasion assay

Cell invasion assay was carried out according to a previous protocol 14. Briefly, cell invasion was evaluated using 8‐μm pore size transwell chambers (Corning, New York, USA). Cells in serum‐free medium were plated in the upper chamber. The medium with 20% FBS was employed as a chemoattractant in the lower chamber. Notably, for cell invasion assay, the upper chamber was coated with Matrigel™ membrane (BD Biosciences, New Jersey, USA). After 24 hrs of incubation, invasive cells on the lower chamber were fixed with paraformaldehyde and stained with crystal violet. The number of cells in nine random microscopic fields was counted under a microscope.

### Immunofluorescence analysis

Cells were fixed with 4% paraformaldehyde for 15 min. and then blocked by incubation in normal goat serum for 30 min. The slides were incubated with primary antibodies: E‐cadherin antibody (1:200, CST, Massachusetts, USA) and Vimentin antibody (1:100, CST, Massachusetts, USA), respectively, at 4°C overnight. Then, the slides were incubated with Texas Red‐conjugated secondary antibody (Sigma‐Aldrich, New Jersey, USA) for 1 hr and counterstained with 4′,6‐diamidino‐2‐phenylindole (DAPI). Finally, images were taken under a fluorescence microscope (Nikon Ti‐S, Japan).

### Cell proliferation assay

Cell counting kit‐8 (CCK‐8, Dojindo, Kumomoto, Japan) assay was performed to evaluate cell viability 14. Briefly, cells were cultured in 96‐well plates at a density 3 × 10^3^ cells per well and were tested at the indicated times (0, 24, 48, 72, 96 hrs) in accordance with the protocol. The absorbance of 450 nm was determined to calculate cell growth rates.

### *In vivo* tumourigenesis

Animal studies were approved by the Ethics Committee of the Fifth Affiliated Hospital of Nantong University and were performed on the basis of the institutional guidelines. Six‐week‐old male BALB/c nude mice were utilized for tumourigenicity. To determine the effect of SPOCK1 on tumour formation *in vivo*, SGC7901 cells (sh‐NC/sh‐SPOCK1) or AGS cells (Vector/SPOCK1) were subcutaneously inoculated into nude mice followed by measurements of tumour size every 5 days. The tumour volume was calculated based on the formula: volume (mm^3^) = (short diameter)^2^ × (long diameter)/2 14. The mice were killed after 30 days, and tumour samples were harvested for tumour mass. To investigate the role of SPOCK1 in tumour metastasis *in vivo*, cells mentioned above were injected intravenously into nude mice *via* the tail vein. At 30 days after the inoculation, the mice were killed, and the removal of the lungs was aimed at counting the number of metastatic nodules in the lungs. Subsequently, harvested lungs were further fixed in formaldehyde for haematoxylin‐eosin staining and IHC staining.

### Statistical analysis

Chi‐square test was performed to analyse the clinicopathologic parameters and correlation between categorical variables. Kaplan–Meier and Log‐rank test were used for survival analysis. The data are presented using mean  ±  S.D. and were analysed by Student's *t*‐test or One‐Way anova. Statistical analyses were conducted using SPSS 21.0 software (SPSS Inc, Chicago, USA). A value of *P* < 0.05 was set as statistical significance.

## Results

### Increased SPOCK1 expression is associated with EMT‑related proteins in gastric cancer

**Figure 1:**
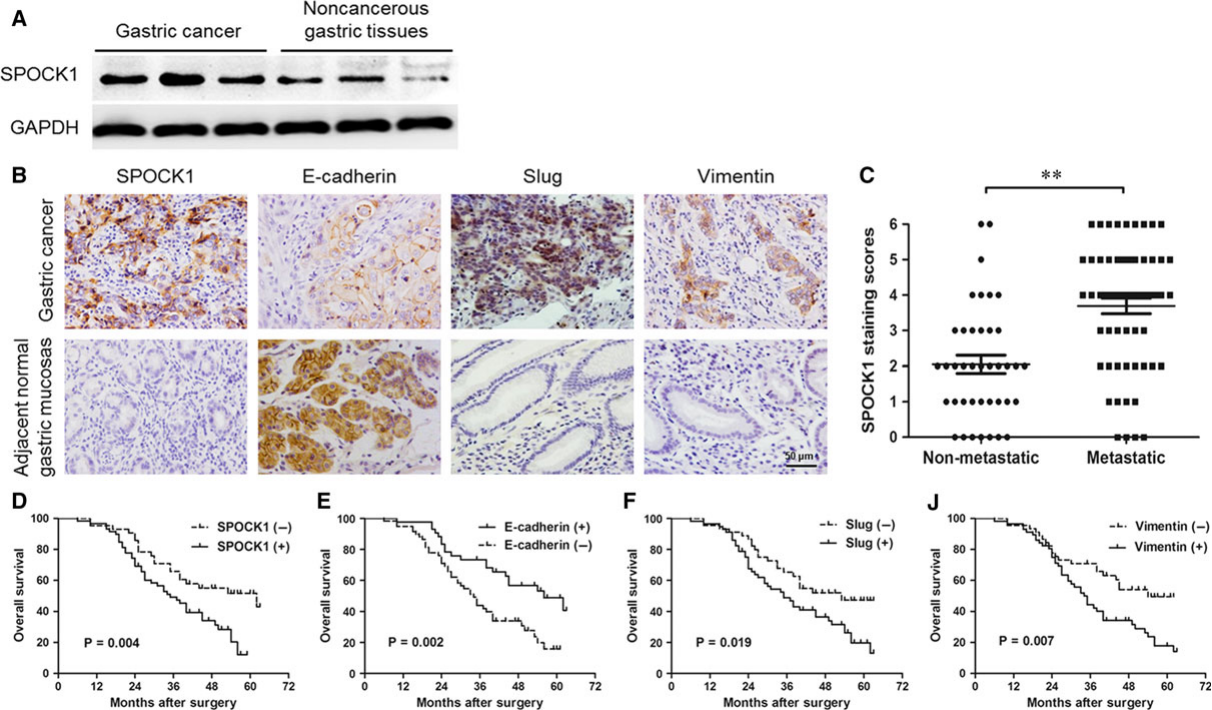
Expressions of SPOCK1 and EMT in gastric cancer and para‐carcinoma tissue, and survival curves. (**A**) Comparison of SPOCK1 expression between gastric cancer and noncancerous gastric tissues by Western blot assay. GAPDH was used as internal control. (**B**) Detection of SPOCK1, E‐cadherin, Slug and Vimentin expressions in gastric cancer tissues and adjacent normal gastric mucosas by immunohistochemical stainings. (**C**) The average staining scores of SPOCK1 expression in patients with or without metastasis. (**D‐J**) Survival curves of gastric cancer patients with SPOCK1, E‐cadherin, Slug or Vimentin expression. **P < 0.001. Scale bar = 50 μm.

To determine the expression level of SPOCK1 in gastric cancer, we initially examined SPOCK1 protein in 40 pairs of gastric cancer tissues (tumour samples and noncancerous gastric samples) by Western blot. Tumour tissues exhibited significantly higher level of SPOCK1 protein than that in noncancerous gastric tissues (Fig. 1A). Besides, the expression levels of SPOCK1, E‐cadherin, Slug and Vimentin were detected in gastric cancer tissues and in adjacent normal gastric mucosas of 102 patients with gastric cancer by immunohistochemical staining (Fig. 1B). As shown in Table 1, SPOCK1, Slug and Vimentin shared markedly higher expression in gastric cancer tissues, compared with those in adjacent normal gastric mucosas (*P* = 0.001, *P* = 0.017 and *P* = 0.001, respectively). Reversely, E‐cadherin exhibited significantly lower expression in gastric cancer tissues (*P* < 0.001). A clinicopathological association study of the 102 patients with gastric cancer demonstrated that the expressions of SPOCK1, E‐cadherin, Slug and Vimentin significantly correlated with T stage, pTNM stage and lymph node metastasis, respectively (*P* < 0.05) (Table 2). Compared with patients without lymph node metastasis, those who developed metastasis exhibited significantly higher staining scores for SPOCK1 (*P* < 0.001) (Fig. 1C), which suggested that SPOCK1 might function importantly in metastasis. More importantly, SPOCK1 expression was found to be related with E‐cadherin, Slug and Vimentin expressions [*P* < 0.001, Contingency coefficient (C) = 0.431; *P* = 0.015, C = 0.234; *P* = 0.005, C = 0.271] in gastric cancer tissues (Table 3). Furthermore, positivity for SPOCK1, Slug or Vimentin expression negatively correlated with post‐operative overall survival in patients with gastric cancer (*P* < 0.05, respectively) (Fig. 1D, F and J). Conversely, positivity for E‐cadherin had the opposite result (*P* < 0.05) (Fig. 1E).

**Table 1:** Expressions of SPOCK1, E‐cadherin, Slug and Vimentin in gastric cancer and corresponding normal gastric mucosas

Proteins	Gastric cancer tissues	Gastric normal mucosa tissues	*P*‐value
SPOCK1			
Positive	59	36	0.001
Negative	43	66	
E‐cadherin			
Positive	43	74	<0.001
Negative	59	28	
Slug			
Positive	57	40	0.017
Negative	45	62	
Vimentin			
Positive	57	33	0.001
Negative	45	69	

**Table 2:** Correlation between SPOCK1, E‐cadherin, Slug and Vimentin expression and clinicopathological features in gastric cancer

Clinicopathological features	*n*	SPOCK1			E‐cadherin			Slug			Vimentin		
		+	−	*P*‐value	+	−	*P*‐value	+	−	*P*‐value	+	−	*P*‐value
Age (yr)													
≥60	69	39	30	0.696	32	37	0.212	38	31	0.812	40	29	0.539
<60	33	20	13		11	22		19	14		17	16	
Gender													
Male	71	37	34	0.076	32	39	0.367	41	30	0.566	38	33	0.467
Female	31	22	9		11	20		16	15		19	12	
Tumor size (cm)													
≥5	61	36	25	0.770	24	37	0.483	35	26	0.711	35	26	0.711
<5	41	23	18		19	22		22	19		22	19	
Lauren's classification													
Diffuse	27	17	10	0.530	13	14	0.462	14	13	0.623	11	16	0.065
Intestinal	75	42	33		30	45		43	32		46	29	
Lymphatic vessel invasion													
With	46	31	15	0.077	16	30	0.172	29	17	0.187	29	17	0.187
Without	56	28	28		27	29		28	28		28	28	
T stage													
T_1_ + T_2_	46	19	27	0.002	25	21	0.024	17	29	<0.001	18	28	0.002
T_3_ + T_4_	56	40	16		18	38		40	16		39	17	
pTNM stage													
I + II	44	20	24	0.027	24	20	0.027	16	28	0.001	18	26	0.008
III + IV	58	39	19		19	39		41	17		39	19	
Lymph node metastasis													
With (N_1_ + N_2_ + N_3_)	62	46	16	<0.001	18	44	0.001	44	18	<0.001	42	20	0.003
Without (N_0_)	40	13	27		25	15		13	27		15	25	

**Table 3:** Correlation analysis among expressions of SPOCK1, E‐cadherin, Slug and Vimentin in gastric cancer tissues by chi‐square test

	SPOCK1				
	Positive	Negative	χ^2^	*P*‐value	C
E‐cadherin					
Positive	13	30	23.241	<0.001	0.431
Negative	46	13			
Slug					
Positive	39	18	5.928	0.015	0.234
Negative	20	25			
Vimentin					
Positive	40	17	8.058	0.005	0.271
Negative	19	26			

C, Contingency coefficient.

### SPOCK1 promotes cell migration and invasion *in vitro*

**Figure 2:**
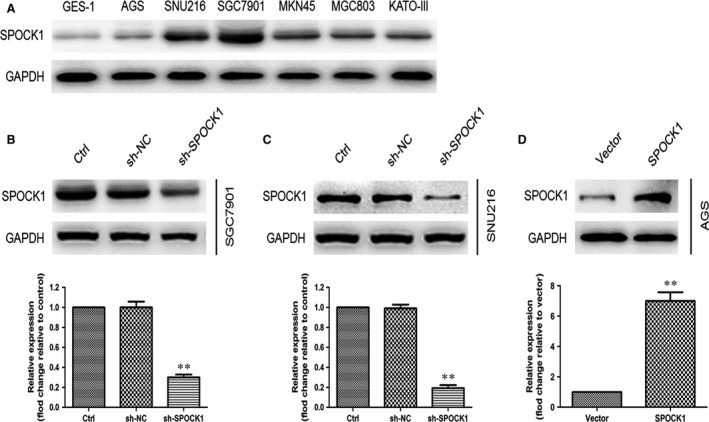
Determination of SPOCK1 expression in gastric cancer cells and normal gastric epithelial cells. (**A**) Relative expression of SPOCK1 protein in gastric cancer cell lines (AGS, SNU216, SGC7901, MKN45, MGC803 and KATO‐III) and normal gastric epithelial GES‐1 cells were measured by Western blot. (**B‐D**) Relative expression of SPOCK1 protein was detected by Western blot in SPOCK1‐knockdown SGC7901 and SNU216 cells and SPOCK1 overexpressing AGS cells. GAPDH was used as internal control. **P < 0.001.

**Figure 3:**
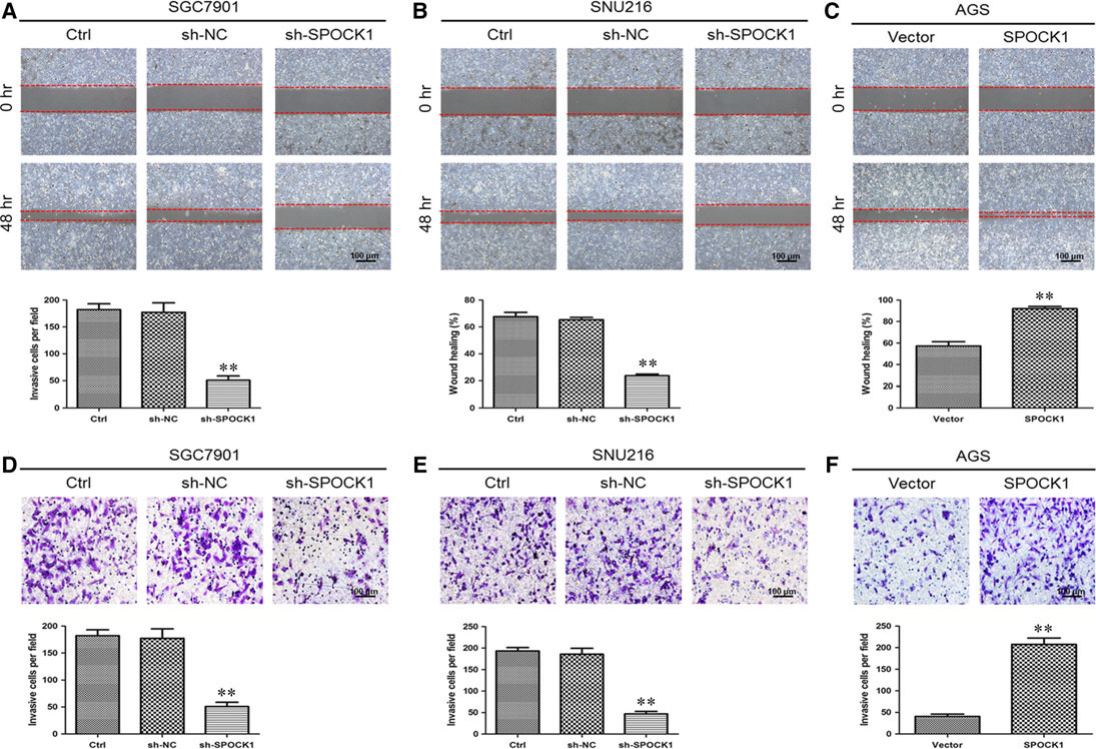
Effects of SPOCK1 silencing and overexpression on gastric cancer cells migration and invasion. (**A**–**C**) Silencing effects of SPOCK1 expression on the migratory capability of SGC7901 and SNU216 cells by wound‐healing assay. (**D**–**F**) Effects of SPOCK1 overexpressing on the invasive ability of AGS cells by transwell invasion assay.**P < 0.001.

To investigate the function of SPOCK1 in the progression of gastric cancer, the endogenous expression of SPOCK1 was determined in six gastric cancer cell lines (AGS, SNU216, SGC7901, MKN45, MGC803 and KATO‐III) and normal gastric epithelial GES‐1 cells. Among them, SPOCK1 expression was highest in SGC7901 and SNU216 cells and was lowest in AGS cells and GES‐1 cells (Fig. 2A), which indicated that SPOCK1 was overexpressed not only in primary tumours but also in gastric cancer cell lines comparing with corresponding normal controls. Then, SGC7901 and SNU216 cell lines were selected for stable transfection using lentivirus shRNA‐mediated reduction of SPOCK1 expression, and AGS cell lines for stable transfection with SPOCK1‐expression vector. Based on the Western blot analysis, we observed that SPOCK1 expression was significantly silenced by shRNA‐SPOCK1 (Fig. 2B and C) and markedly elevated by SPOCK1 overexpression (Fig. 2D). To further assess the effects of SPOCK1 on the migration and invasion of gastric cancer cells *in vitro*, the wound‐healing assay and transwell invasion assay were employed. SPOCK1 silencing reduced the migration capability of SGC7901 and SNU216 cells (Fig. 3A and B), while SPOCK1 overexpression enhanced the migration capability of AGS cells (Fig. 3C). Consistently, the transwell invasion assays indicated that SPOCK1 knockdown decreased the invasive capability of SGC7901 and SNU216 cells (Fig. 3D and E), while overexpression of SPOCK1 in AGS cells demonstrated the opposite effect (Fig. 3F). These results suggest that SPOCK1 facilitates gastric cancer cells migration and invasion *in vitro*.

### SPOCK1 promotes cell invasion through Slug‐mediated EMT

**Figure 4:**
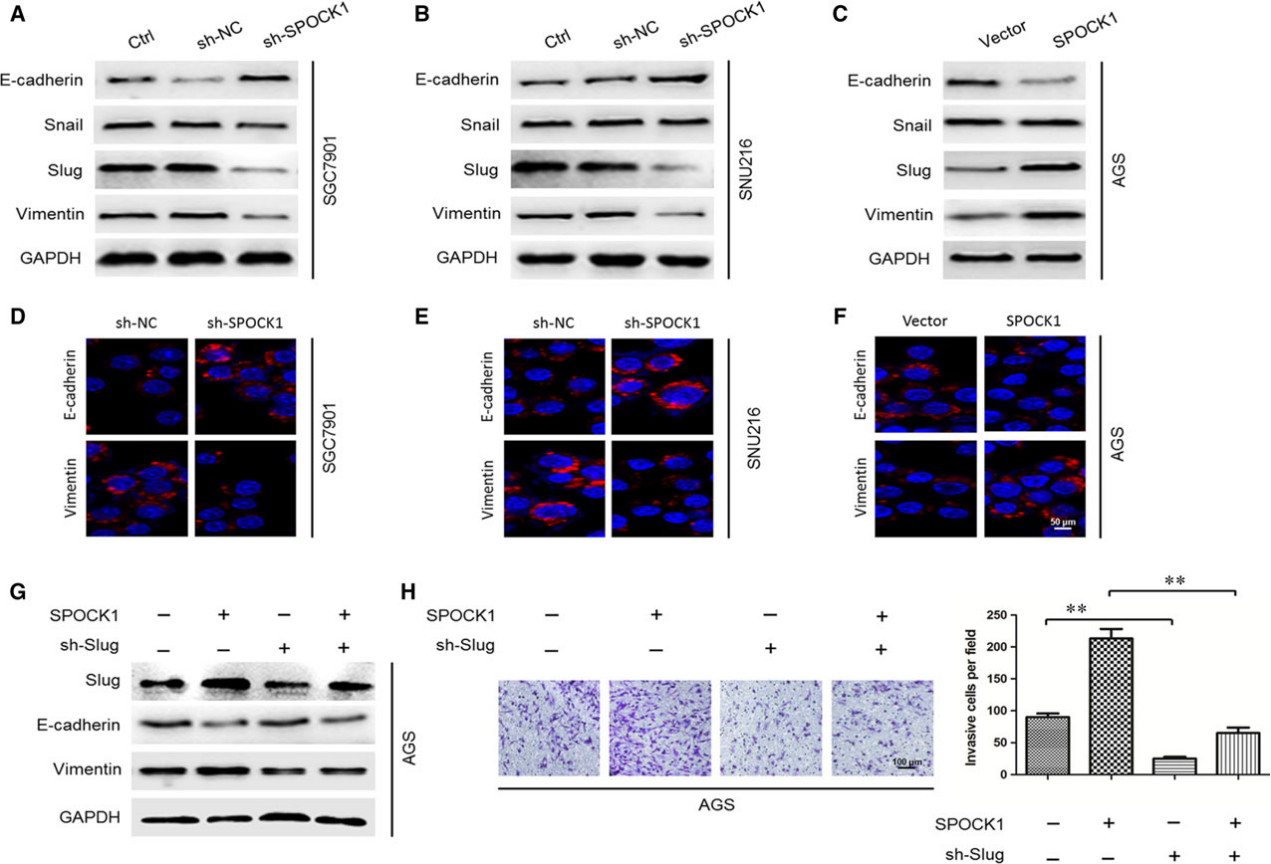
SPOCK1 induced cell EMT and invasion *via* a Slug‐dependent mechanism. (**A,B,D,E**) Influences of SPOCK1‐depletion on EMT‐related markers in SGC7901 and SNU216 cells. (**C,F**) Effects of SPOCK1 overexpressing on EMT‐related markers in AGS cells. (**G**–**H**) Insight into a Slug‐dependent mechanism of SPOCK1‐inducing cell EMT and invasion, by Western blot and transwell assay. GAPDH was used as internal control. **P < 0.001.

To characterize whether SPOCK1 enhanced the invasiveness of gastric cancer cells through EMT processes, the EMT biomarkers (E‐cadherin and Vimentin) and transcription factors (Snail and Slug) were determined by Western blot analysis and immunofluorescence analysis. We found that E‐cadherin expression was increased in SPOCK1‐depleted SGC7901 or SNU216 cells, coupled with a noticeable decrease in the expressions of Vimentin and Slug (Fig. 4A, B, D and E). Conversely, overexpression of SPOCK1 in AGS cells reversed this phenotype (Fig. 4C and F). However, the expression of Snail was not significantly changed in SGC7901, SNU216 and AGS cells (Fig. 4A–C). To evaluate the role of Slug in SPOCK1‐induced EMT and cell invasion, the transwell invasion assay and Western blot analysis were performed. It was revealed that shRNA‐mediated suppression of Slug in AGS cells resulted in a substantial reversal of SPOCK1‐induced EMT (Fig. 4G). Likewise, shRNA‐mediated knockdown of Slug in AGS cells led to a significant inhibition of basal invasion. Besides, sh‐Slug expression in AGS cells remarkably suppressed SPOCK1‐induced cell invasion (Fig. 4H). These results indicate that SPOCK1 promotes cell invasion, at least in part, through a Slug‐dependent EMT mechanism.

### SPOCK1 facilitates cell invasion and metastasis *in vivo* by inducing EMT

**Figure 5:**
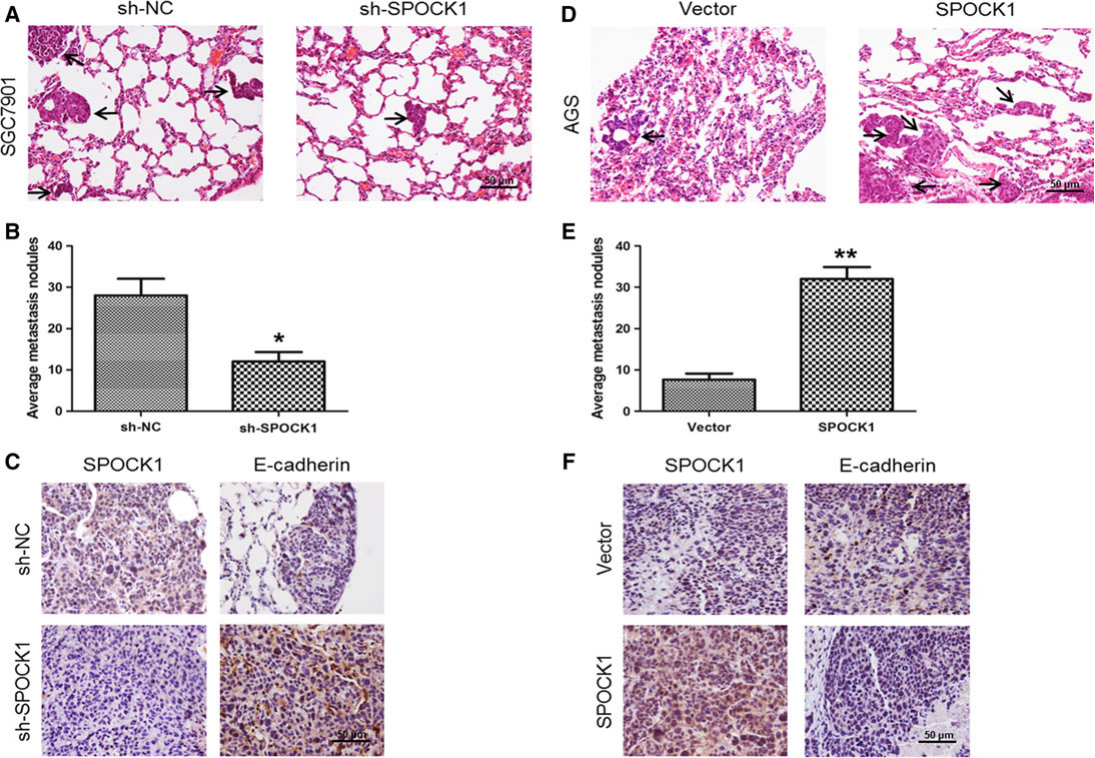
Influences of SPOCK1 silencing and overexpression on the metastasis of gastric cancer cells *in vivo*. (**A,D**) Lung metastatic nodules (indicated by black arrows) were histologically observed through HE‐stained samples. (**B,E**) The number of average lung metastatic nodules was calculated. (**C,F**) Expressions of SPOCK1 and E‐cadherin in lung metastatic nodules were determined by immunohistochemistry. *P < 0.05, **P < 0.001. Scale bar = 50 μm.

To confirm the role of SPOCK1 in the invasion and metastasis of gastric cancer cells *in vivo*, we performed a lung metastasis model through the injection of tail vein in nude mice. We found that a significantly less number of lung metastasis foci could be observed in the sh‐SPOCK1 group, compared with the sh‐NC group in SGC7901 cells at 30 days after injection (Fig. 5A and B). Inversely, mice injected with SPOCK1‐overexpressing AGS cells exhibited more metastatic nodules compared with the Vector group (Fig. 5D and E). Additionally, consistent with the results *in vitro*, the sh‐SPOCK1 group in SGC7901 cells exhibited lighter staining of SPOCK1 and stronger staining of E‐cadherin, compared with sh‐NC group according to the immunohistochemical analysis (Fig. 5C), while overexpression of SPOCK1 in AGS cells showed the opposite effect (Fig. 5F). These results demonstrate that SPOCK1 facilitates the invasion and metastasis of gastric cancer cells *via* inducing EMT.

### Effects of SPOCK1 expression on cell proliferation *in vitro* and *in vivo*

**Figure 6:**
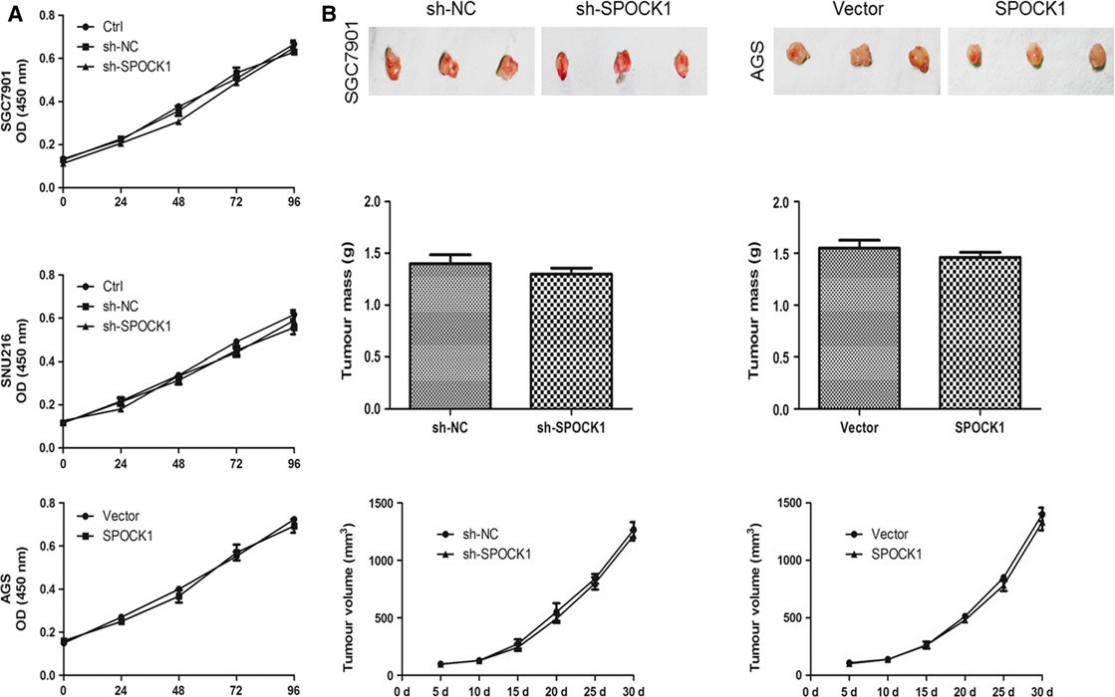
Effects of SPOCK1 knockdown and overexpression on gastric cancer cells proliferation. (**A**) Effects of SPOCK1 knockdown and overexpression on the proliferation abilities of gastric cancer cells (SGC7901, SNU216 and AGS) *in vitro* by CCK‐8 assay. (**B**) Influences of SPOCK1 knockdown and overexpression on the proliferation abilities of gastric cancer cells (SGC7901 and AGS) *via* the subcutaneous injection of the indicated cells into nude mice, as well as the measurement of tumour mass and tumour volume.

To exclude the possibility that the influence of SPOCK1 on cell migration and invasion was as a result of different cell proliferation rates, the cellular growth rates *in vitro* were compared. We found that all cells showed similar proliferation rates *in vitro* by CCK‐8 assay (Fig. 6A). Similarly, to ascertain whether SPOCK1 could exert an influence on tumour formation *in vivo*, SGC7901 cells (sh‐NC and sh‐SPOCK1) or AGS cells (Vector and SPOCK1) were inoculated subcutaneously into nude mice develop implant tumour, respectively. Obviously, the weight and the volume of implant tumour were almost identical between the two groups (Fig. 6B). These results showed that SPOCK1 is not essential for cell proliferation, which further make obvious the phenomenon that SPOCK1‐induced cell invasion and metastasis is not associated with cellular proliferation rate *in vitro* and *in vivo*.

## Discussion

Metastasis is still a crucial impediment to the effective treatment for patients with gastric cancer, despite significant progress has been made in surgical care and chemotherapy over the past decades 3. Identification of novel targeted molecules and the underlying mechanism(s) involved in cell motility and invasion contributes to an in‐depth understanding of cancer metastasis, with the purpose of detection of novel therapeutic approaches. Recently, accumulated evidence demonstrated that SPOCK1 played a key role in cell proliferation and invasion in various types of cancer 16, 17. Notably, an investigation revealed that SPOCK1 was overexpressed in gastric cancer tissues, by IHC analysis using tissue microarrays on a large number of patients, suggesting that SPOCK1 could become a clinically useful candidate if more attention paid to its diagnostic, prognostic and therapeutic value 18. Interestingly, Kim 19 first revealed that SPOCK1‐mediated EMT signalling conferred acquired resistance to lapatinib in human epidermal growth factor receptor 2 (HER2)‐positive gastric cancer. To our knowledge, the effect of SPOCK1 expression on the invasion and metastasis of gastric cancer has not yet been fully addressed.

In this study, the clinical association analysis demonstrated that SPOCK1 was overexpressed in gastric cancer tissues compared with that in para‐carcinoma tissues, and significantly associated with clinical metastasis, EMT markers and an unfavourable prognosis in a panel of patients with gastric cancer, indicating that SPOCK1 may function in gastric cancer progression. Additionally, the *in vitro* and *in vivo* assays indicated that SPOCK1 facilitated the invasion and metastasis of gastric cancer *via* Slug‐mediated EMT, and yet made no difference to the proliferation of gastric cancer cells, suggesting its key role in cancer cell invasion and metastasis.

Metastasis is an important cause of cancer‐related deaths in patients. Strikingly, EMT is integral in cancer progression and plays a pivotal role during cancer metastasis. During EMT, epithelial cell loses their characteristics, reprogrammes gene expression and gains mesenchymal properties, which enable the evolution of an invasive phenotype and increase the migratory capability of individual cancer cell 6. Consequently, EMT has been regarded as the full realization of cancer cell invasive behaviour. It is interesting to note that SPOCK1 facilitated the migration and invasion of cancer cells *via* the mechanism of EMT in certain types of cancers, such as gallbladder cancer 13 and lung cancer 20. Besides, during the process of SPOCK1‐inducing EMT, the cooperation and crosstalk between translational regulation and signalling pathways were also further revealed. For example, in the investigation of SPOCK1‐mediated cells invasion and metastasis in gallbladder carcinoma, the transcription factor Snail was a key switch in the transformation of cell invasion behaviour controlled by SPOCK1‐inducing EMT 13. Similarly, PI3K/AKT and Wnt/β‐catenin signalling pathways were also found involved in the advancement of glioma cancer 21. In the current study, regarding the mechanism of SPOCK1‐mediated EMT in gastric cancer invasion and metastasis, for the first time possibly we explored the roles of Snail and Slug in the SPOCK1‐mediated EMT. Our data revealed that SPOCK1 facilitated cells invasion and metastasis through activation of Slug rather than Snail, subsequently leading to the EMT evolution. And knockdown of Slug expression could reverse the process, implying that Slug played an essential role in SPOCK1‐mediated EMT. Interestingly, it is worth to note that PI3K/AKT signalling pathway was involved in SPOCK1‐mediated EMT in gallbladder cancer 13 and glioma cancer 21. Hence, in the next step, whether the PI3K/AKT signals involved in SPOCK1‐induced EMT in the invasion and metastasis of gastric cancer deserves further investigation.

Beyond all that, though, as previously reported, SPOCK1 could promote cell proliferation in colorectal cancer 15 and prostatic cancer 16. However, in this study of ours, to exclude the possibility that the positive role of SPOCK1 in cell migration and invasion was as a result of different proliferation rates, our data showed that SPOCK1 almost did not affect the proliferation of gastric cancer cells *in vitro* and *in vivo* assays. Otherwise, it is necessary to notice that the construction of mice metastatic lung nodules *via* the inoculation of cancer cells into the tail vein of nude mice intravenously did not follow the principle of physiological metastatic model in this study. Yet, it could imitate the overflow of cancer cells from blood vessels into the target organ, considered as a crucial step in the metastatic process 22. More importantly, this model has been employed to analyse the metastatic potential of cancer cells in previous studies 23, 24.

Interestingly, in addition to what has already been mentioned above, a recent study revealed that *H. pylori* HP0175 protein elicited a peculiar Th17 inflammation which, if long‐lasting and unabated, may represent an immunopathological condition that links the infection and gastric cancer, suggesting that the Th17 pathway and HP0175 may contribute to malignancy and tumour invasion to some extent. Besides, increased proportions of Th17 cells were present in tumour‐draining lymph nodes of patients with advanced disease 25. Therefore, the further exploration of the relationship among SPOCK1, the Th17 pathway and HP0175 may be very meaningful.

In conclusion, this study of ours revealed for the first time that the high expression of SPOCK1 was closely correlated with cancer metastasis and patient survival, and SPOCK1 promoted the invasion and metastasis of gastric cancer cells *via* Slug‐mediated EMT, thus possibly providing a novel therapeutic target for gastric cancer. Nevertheless, it is worth exploring the more in‐depth mechanism(s) involved in the complex interaction of SPOCK1 and Slug.

## Trial registration

This study was approved by the Ethics Committee of the Fifth Affiliated Hospital of Nantong University (No. 201101205).

## Conflict of interest

The authors confirm that there are no conflicts of interest.
